# Book Review: English Medium Instruction: Content and Language in Policy and Practice

**DOI:** 10.3389/fpsyg.2021.686997

**Published:** 2021-06-02

**Authors:** Liyan Huang

**Affiliations:** Center for Language Cognition and Assessment, School of Foreign Studies, South China Normal University, Guangzhou, China

**Keywords:** english medium instruction, content learning, second language acquisition, EMI, educational policy

The world has seen a rapid development of English Medium Instruction (EMI) since the turn of the century, with a number of studies on EMI published over the last 20 years. According to Ernesto Macaro, EMI refers to “The use of the English language to teach academic subjects (other than English itself) in countries or jurisdictions where the first language (L1) of the majority of the population is not English” (Macaro, 2018, p. 19). To the best of my knowledge, this is the first single authored book that attempts to approach the EMI phenomenon from a global perspective, covering studies in both secondary and tertiary education.

The book has 10 chapters, mainly discussing EMI terminology and definition, language policies, key participants in EMI, classroom instruction, and learner strategies. In Chapter 1, Macaro examines the origins and implementation of different terminology and programs somehow related to EMI and proposes a continuum, with “language-dominant objectives” (Immersion Education) at one end and “content-dominant objects” (EMI) at the other to mirror English L2 classrooms around the world. Chapter 2 includes a systematic review of how the supranational and country-specific language policies in the European Union, the Association of Southeast Asian Nations, and the Gulf Cooperation were formulated and implemented. Some trends can be discerned, e.g., that EMI is the result of the globalization of economic structures and the internationalization of education.

The subsequent chapters cover the topics of EMI stakeholders, the teaching process and its impact. In Chapters 3 and 4, Macaro explores the beliefs of teachers and students both at the secondary and tertiary level. The vast majority of studies show that teachers can be categorized into four groups: “active promoters,” “consenting participants,” “passive victims,” and “resistance fighters” of EMI. The analyses of the 21 studies related to students' attitudes toward EMI indicate that the beliefs and attitudes of students match those of teachers. Chapters 5 and 7 tackle the main elements of the teaching process, i.e., language of instruction and classroom interaction. Macaro discusses the phenomenon of “World Englishes” and “English as a Lingua Franca,” what variety of English should be taught or used, and who should teach in EMI classes. In addition, he applies SLA interaction theory to demonstrate how language is being used in EMI classrooms. Chapters 8 and 9 are relevant to the impact of EMI on teacher education and learner strategies. He expounds learner strategies theory in SLA, particularly listening and vocabulary comprehension strategies, and discusses the use and development of strategies in EMI classrooms. A cost-benefit analysis of EMI is made in Chapter 6 and revisited in the last chapter. Macaro identifies three drivers of EMI and urges that a stakeholder research-driven approach to EMI should be adopted, instead of a top-down one.

EMI, as a global phenomenon, is a relatively new field of research and there is a lack of consensus on EMI terminology and definition. With this volume, Macaro has increased our understanding of the concept of EMI by proposing an innovative continuum. According to Macaro, EFL can be referred to as the teaching of “General English,” mainly aimed at language learning. The prime objectives of EAP and ESP are also language development, but in academic or occupational settings. With regard to CLIL and EMI, he points out that it is crucial to separate these two approaches from CBI, which targets students whose L2 is their dominant language. CLIL and EMI students are taught via an L2 which is not the language of the majority of the population around them. CLIL integrates content learning with language teaching, while EMI aims at teaching academic subjects other than language itself. He also points out that it is hard to define EMI for many reasons and the definition he proposes is still open to challenge. Furthermore, Macaro has built a bridge between the research fields of Applied Linguistics and Education, which proposes a new perspective of research into the phenomenon of EMI. For example, codeswitching is common in EFL or EMI classroom interaction. Some research suggests that content teachers do not have a principled approach to L1 use and that they are even unaware of their own switching between L2 and L1. Concerning learners' strategies in an EMI context, Macaro claims that there is no need to differentiate “language use tasks” for EFL and EMI. Thus, strategies used in accomplishing tasks in EFL contexts could be applied in EMI. The EMI strategy research area is still in its infancy and researchers could make reference to SLA strategy research, which has a long-standing tradition.

Empirical studies carried out by researchers from a total of 43 countries around the world were analyzed in the volume, which can inform current practice in many ways. Macaro succeeds in unpacking the complexities of EMI practice from different perspectives, e.g., the launch of and reflection on policies, comparison between teacher and student beliefs and attitudes, characteristics of classroom discourse and interaction patterns, teacher professional development, learning processing in EMI classrooms, etc.

It would be more comprehensive if the book could include an in-depth discussion of learning mechanisms in EMI classes, e.g., learners' cognitive processing while working on content tasks using English, and learning outcome assessment. Notwithstanding, this is a valuable volume which provides administrators, researchers, teachers, and students in the fields of Education, Applied Linguistics, and EMI in particular with an illuminating insight into the phenomenon of EMI. It is extremely valuable to EMI policy making, classroom research, teacher professionalization, etc. It can also facilitate our reflection on current practice and offer us a number of thought-provoking questions for further research.

## Author Contributions

The author confirms being the sole contributor of this review and approved it for publication.

## Conflict of Interest

The author declares that the research was conducted in the absence of any commercial or financial relationships that could be construed as a potential conflict of interest.
